# Occurrence and Characteristics of Livestock-Associated Methicillin-Resistant *Staphylococcus aureus* in Quarter Milk Samples From Dairy Cows in Germany

**DOI:** 10.3389/fmicb.2019.01295

**Published:** 2019-06-12

**Authors:** Kristina Kadlec, Monika Entorf, Thomas Peters

**Affiliations:** ^1^Institute of Farm Animal Genetics, Friedrich-Loeffler-Institut, Mariensee, Germany; ^2^Dairy Herd Consulting and Research Company (MBFG), Wunstorf, Germany

**Keywords:** staphylococci, antimicrobial resistance, *mecA*, oxacillin resistance, mastitis, Germany, dairy, animal

## Abstract

The aim of this study was to identify and characterize LA-MRSA in a very recent collection of staphylococci isolated from bovine quarter milk samples. All milk samples (*n* = 14,924) sent to the MBFG in March 2017 were included into this study. The samples originated from 3,887 cows with 3,367 samples from 2,280 animals being positive for bacteria, prototheca and/or yeast. The second most common infectious agent was *Staphylococcus* and 659 isolates were investigated. *Staphylococcus aureus* was confirmed by PCR for the *spa* gene. A CC398-specific PCR was performed for all *S. aureus* isolates. Susceptibility to penicillin was tested for all isolates by agar disk diffusion. All oxacillin resistant isolates were analyzed by microarray and tested for their susceptibility to 30 antimicrobial agents. Of the isolates 372 were *S. aureus* from Germany with 214 isolates being not epidemiologically related. Among the independent isolates nine were identified as oxacillin resistant. In addition five isolates epidemiologically related to these nine were MRSA. One of them showed differences to the other MRSA isolate from the same farm resulting in altogether ten different MRSA isolates. All ten belonged to the clonal complex CC398. These ten LA-MRSA isolates had three to six antimicrobial resistance genes. The gene *mecA* was in all cases located on a SCC*mec* V element. Among the remaining *S. aureus* seven independent isolates belonged to CC398. In conclusion this study showed a high detection rate of staphylococci in bovine quarter milk samples. In contrast MRSA was rarely detected and belonged in all cases to CC398. Only 7/214 MSSA (3.3%) belonged to this CC.

## Introduction

Staphylococci belong to the most important bacterial causes of mastitis in dairy cattle worldwide. Among the isolates identified during routine diagnostics on milk samples in Germany the resistance situation is still favorable, but the number of resistant staphylococci seems to be increasing (GERM-Vet, 2016). The same situation is seen worldwide as documented for example for Europe (de Jong et al., 2018). Among them, methicillin-resistant *Staphylococcus aureus* (MRSA) are seen frequently. Far less is known about methicillin-susceptible *S. aureus* (MSSA) in livestock. Very commonly these MRSA belong to the clonal complex 398 (CC398), known as livestock-associated MRSA (LA-MRSA). Such type of MRSA isolates is also present in other animals, is frequently isolated from human samples and can cause infections in human patients (Cuny et al., 2013). Moreover, it has been observed, that especially LA-MRSA are able to acquire antimicrobial resistance genes as well as genes coding for tolerance to heavy metals or disinfectants (Kadlec et al., 2012; Wendlandt et al., 2013). In contrast to other staphylococci and other CCs from *S. aureus*, LA-MRSA gain multiresistance by the acquisition of large multiresistance plasmids and not so often by the presence of several small plasmids (Feßler et al., 2018). Studies on the characterization of MRSA isolates from bovine mastitis identified cows as hosts of this type of MRSA (Feßler et al., 2010; Vanderhaeghen et al., 2010). Since then, LA-MRSA has been identified in bovine milk samples in several countries and on several continents. A number of studies had a look into the characteristics of such MRSA of bovine origin, but information is still scarce (Nemeghaire et al., 2014; Liu et al., 2017; Klibi et al., 2018; Ronco et al., 2018).

The aims of this study were (i) to gain insight into presence of MRSA among isolates from milk samples from cows, (ii) to identify the occurence of LA-MRSA and LA-MSSA, and (iii) to characterize LA-MRSA isolates collected in 2017 in Germany.

## Materials and Methods

In this study all milk samples (*n* = 14,924) send to the MBFG in March 2017 were included. MBFG is among the diagnostic laboratories in Germany with the highest number of milk samples analyzed. MBFG is located in the northern part of Germany, but receives samples from all over the country as well as from neighboring countries. The quarter milk samples were sent for bacteriological analysis. In most of the cases a milk sample is sent from each lactating quarter, thus four samples per cow. In the majority of cases at least one of the quarters showed a clinical mastitis. Other reasons for sending milk samples are subclinical mastitis, control after mastitis treatment or a control prior to drying off. The laboratory procedure includes direct plating on sheep blood agar without any enrichment or selective steps. The milk samples originated from 3,867 cows with 3,367 samples from 2,280 animals being positive for bacteria, prototheca and/or yeast. Second most common infectious agent was *Staphylococcus* and 659 isolates were investigated. The isolates originated from 291 dairy farms (Table 1). While 166 *Staphylococcus* isolates were from farms which contributed only one isolate each, 60 isolates came from 30 other farms which accounted for two *Staphylococcus* isolates each and 22 farms contributed three *Staphylococcus* isolates. The remaining 307 isolates originated from 43 farms which sent in four or more milk samples being positive for staphylococci. The samples from the dairy farms were sent in partly one order and partly on different days. The farm which contributed the highest number of *Staphylococcus* isolates had sent in samples on 8 days and 31 staphylococci were detected in the samples from 21 different animals.

**TABLE 1:** Sample collection from routine diagnostics in March 2017.

**Collection**	**Number**			
	**In total**	**Positive for bacteria, prototheca or yeast**	**Independent *S. aureus***	**Positive for LA-MRSA**
Number of milk samples	14,924	3,367	214	12
Number of cows	3,887	2,280	214	11
Number of farms	n.d.	n.d.	214	9

*n.d., not determined.*

Within the framework of the study *Staphylococcus* isolates were confirmed as *S. aureus* by a PCR for the *spa* gene. The PCR used for this approach was the one used for *spa* typing (Kadlec et al., 2009). In isolates negative in this PCR a part of the 16s rDNA was amplified by another PCR, the product sequenced and compared via blastN (Weiß et al., 2013). Susceptibility to penicillin was tested by agar disk diffusion with 10U penicillin G disks and susceptibility to oxacillin by broth dilution according to the Clinical and Laboratory Standards Institute (CLSI, 2018). The *S. aureus* strains ATCC 25923 as well as ATCC 29213 were used as quality control, respectively (CLSI, 2018). Moreover, MICs to ciprofloxacin (16–0.008 mg/L), clindamycin (64–0.03 mg/L), enrofloxacin (16–0.008 mg/L), erythromycin (32–0.015 mg/L), gentamicin (256–0.12 mg/L), kanamycin (128–0.03 mg/L), linezolid (64–0.03 mg/L), oxacillin (8–0.0015 mg/L), pirlimycin (64–0.03 mg/L), quinupristin/dalfopristin (32–0.015 mg/L), tetracycline (256–0.12 mg/L), trimethoprim/sulfamethoxazole (SXT) (32/608–0.015/0.3 mg/L), trimethoprim (256–0.12 mg/L), and sulfamethoxazole (1024–1 mg/L) were determined by broth dilution for the oxacillin-resistant isolates. The oxacillin-resistant isolates were characterized by the staphytype microarray (Monecke et al., 2011). Presence of antimicrobial resistance genes was tested by previously described PCR assays or by the staphytype microarray (Feßler et al., 2010).

## Results

Among the 659 *Staphylococcus* isolates, 372 were *S. aureus*. In some cases *S. aureus* was isolated from more than one quarter milk sample of the respective cow. In some other cases *S. aureus* was isolated from more than one animal in the same herd. Such isolates were also not considered as epidemiologically independent. Finally, this collection comprised 214 epidemiologically independent *S. aureus* isolates. In addition, in ten cases in which *S. aureus* isolates were considered as epidemiologically related to each other, the isolates showed differences in their characteristics. For example we had cases, in which in one quarter sample from a cow MRSA was found and in another sample from the same animal MSSA was identified (Table 2).

**TABLE 2:** Characteristics of the 10 LA-MRSA isolates (CC398, SCC*mec* type V) from dairy cows from Germany.

**Isolate ID**	**Date**	**Other cows with staphylococci from the same farm**	**Resistance phenotype^a^**	**Resistance genotype**	**Differences in the microarray^b^**						
					***agrB***	***agrC***	**ssl01/set6**	**ssl05/set3**	***sdrC***	***mprF***
Wu65	2017-03-03	2 cows 1: MRSA (*n* = 2, Wu65) 2: MRSA	BLA, TET	*mecA*, *blaZ*, *tet*(K), *tet*(M)	+	+	+	+	+	+
Wu69-1	2017-03-03	2 cows 1: MRSA (Wu69-1), *S. succinus* (same quarter) 2: MSSA (*n* = 2)	BLA, TET	*mecA*, *blaZ*, *tet*(K), *tet*(M)	+	+	+	−	+	+
Wu116	2017-03-04	2 cows 1: MRSA (*n* = 2, Wu116), *S. simulans* 2: MSSA	BLA, TET	*mecA*, *blaZ*, *tet*(K), *tet*(M)	+	+	+	−	+	+
Wu250	2017-03-03	3 cows 1: MRSA (Wu250) 2: *S. chromogenes* 3: MSSA (other cow, 2017-03-16)	BLA, TET	*mecA*, *blaZ*, *tet*(K), *tet*(M)	+	−	+	−	−	−
Wu306	2017-03-15	3 cows 1: MRSA (Wu306) 2: MSSA 3: *S. chromogenes*	BLA, TET, ML, SPE	*mecA*, *blaZ*, *tet*(K), *tet*(M), *erm*(A), *spc*	+	+	+	−	+	−
Wu488	2017-03-24	7 cows 1: *S. chromogenes* 2: MRSA (Wu488), MSSA (CC398) 3: *S. chromogenes* 4: MRSA (*n* = 2, Wu490), MSSA (CC398) 5: *S. simulans* 6: MSSA 7: MSSA	BLA, TET, ML, TMP, SUL, SPE	*mecA*, *blaZ*, *tet*(M), *tet*(L), *erm*(A), *dfrK*, *spc*	+	+	+	−	+	+
Wu490	2017-03-24	cf. Wu488	BLA, TET, ML, TMP, SUL, SPE	*mecA*, *blaZ*, *tet*(K), *tet*(L), *erm*(A), *dfrK*, *spc*	+	−	+	−	?	−
Wu609	2017-03-29	9 cows 1: MSSA (*n* = 3) 2: MSSA (*n* = 2) 3: MRSA Wu609 4: MSSA 5: MSSA, *S. haemolyticus* 6: *S. rostri* 7: MSSA (*n* = 2) 8: MSSA (*n* = 2) 9: MSSA (*n* = 3)	BLA, TET	*mecA*, *tet*(K), *tet*(M)	+	+	+	−	+	+
Wu640	2017-03-30	5 cows 1: MSSA 2: MSSA 3: MSSA (*n* = 2, CC398) 4: MRSA Wu640, MSSA 5: MSSA (*n* = 3)	BLA, TET, ML, TMP, SUL, SPE	*mecA*, *blaZ*, *tet*(K), *tet*(M), *erm*(A), *spc*	+	+	+	−	+	−
Wu658	2017-03-31	1 cow: MRSA Wu658	BLA, TET	*mecA*, *blaZ*, *tet*(M)	−	−	−	−	−	−

*^a^BLA, β-lactams; TET, tetracycline; ML, macrolides/lincosamides; TMP, trimethoprim; SUL, sulfonamides; SPE, spectinomycin. ^b^?, an ambiguous results was seen in the microarray, it is unclear whether the isolate harbors this gene or not.*

Susceptibility testing revealed 9/214 oxacillin resistant epidemiologically independent isolates. In addition, five of the epidemiologically related isolates were resistant to oxacillin. In three cases the same animal had a second indistinguishable MRSA in a different quarter sample. In one case the same MRSA was identified in a sample from a different cow. Two indistinguishable MRSA isolates were seen in two animals from the same farm in the fifth case (isolate Wu490), resulting in a total of 10 different MRSA isolates (Table 2). All ten isolates were tetracycline resistant with slight differences in tetracycline resistance genes: Two and one isolates lacked *tet*(K) or *tet*(M), respectively, whereas two isolates carried *tet*(L). Four MRSA were macrolide and lincosamide resistant encoded by *erm*(A). All four had a very high spectinomycin MIC and harbored the resistance gene *spc*. Three of these four isolates were also trimethoprim and sulfonamide-resistant. In addition to *mecA*, all MRSA isolates carried *blaZ* (Table 2).

All MRSA were positive in the PCRs for CC398. The microarray underlined this finding and classified them also as MRSA CC398. All MRSA isolates had the methicillin resistance gene *mecA* on a SCC*mec* element type V. All isolates had capsule type 5 and *agrI*. Enterotoxin genes were not detected in the LA-MRSA isolates. The ten MRSA isolates varied only in a few of the genes, which are included in the microarray (Table 2).

Among the ten MRSA CC398, one isolate originated from a farm in which no other staphylococci were detected. The remaining MRSA CC398 were from farms in which staphylococcal isolates were isolated from two to nine cows. In one case (isolate ID: Wu69-1) the MRSA was isolated from the same quarter as a *Staphylococcus succinus*, which was methicillin-susceptible.

In addition to LA-MRSA, seven of 214 (3.3%) independent isolates and 11/358 (3.1%) isolates in total among the methicillin-susceptible *S. aureus* isolates belonged to CC398 according to the PCR. Three of these were from farms with LA-MRSA (Table 2). In two cases the LA-MSSA isolate was identified from the same cow, but from a different quarter than the LA-MRSA, in the remaining case two indistinguishable LA-MSSA were isolated from different quarters from a cow without LA-MRSA.

## Discussion

In total 214 independent *S. aureus* isolates of the 372 isolates form Germany were studied in detail. It seems to be common knowledge, that *S. aureus* in dairy cattle is a herd health problem and that *S. aureus* spreads within the farm. Insights into single farms underline this assumption (Feßler et al., 2012). From a number of dairy farms more than one *S. aureus* was isolated. In general all isolates from the same farm were not considered as independent. For the counted numbers we considered such isolates as being epidemiologically related. However, we saw even single animals carrying two different *S. aureus*. Based on the epidemiological relatedness, only selected isolates were tested for their susceptibility. Therefore it cannot be excluded, that more isolates would show differences to isolates presumed to be related. Among all isolates tested for their susceptibility, ten isolates showed different susceptibility profiles compared to isolates considered as epidemiologically related. These ten isolates were also included into further tests. Moreover, our decision was based solely on susceptibility profiles, further in-depth analysis could also reveal differences between *S. aureus* isolates from the same animal or the same farm (Kadlec et al., 2015). In general this is a limit of most of the studies performed. It is definitely a waste of resources to investigate copy isolates, but it is unknown how high the number of animals is, that carry a mix of bacteria belonging to the same species. During routine diagnostics from a culture consisting of a single species a single colony is picked and used for further analysis. In our study, however, we saw isolates with different characteristics from the same quarter, the same animal or the same farm. This study confirms, the presence of different *S. aureus* isolates on one farm and the presence of different *S. aureus* isolates in different quarters of the udder of the same animal. It suggests the possibility of the presence of different *S. aureus* in the same sample in very rare cases.

The identification of 9/214 independent isolates as oxacillin-resistant clones fits well into the data form the German National Resistance monitoring program GE*RM*-Vet, investigating a representative German collection. In GE*RM*-Vet nine of 205 *S. aureus* isolates collected in 2013/2014 were identified and confirmed as MRSA (GERM-Vet, 2016). It is also in accordance with the identification of 19/430 isolates from Belgium (Bardiau et al., 2013). However, in the latter study one of the MRSA isolates did not belong to CC398. As in this study only CC398 MRSA were identified among isolates from Switzerland (Sakwinska et al., 2011). Whereas MRSA from bovine mastitis most commonly belong to CC398, MSSA from cows belong to other CCs (Holmes and Zadoks, 2011). This was also seen in the present study with only 3% of the MSSA belonging to CC398. Although the number of MRSA CC398 was low in this study, the finding of such isolates is still not so common and worthwhile to mention. In general the rate of MRSA among isolates from bovine mastitis is still low, but seems to be increasing and should be monitored.

The resistance phenotypes and genotypes resembled those previously seen in LA-MRSA (Kadlec et al., 2012). However, this study also underlines that in isolates from mastitis you still find a favorable resistance situation. This is obvious by two results of the study: (1) The number of MRSA was low. (2) Six of the ten different LA-MRSA isolates were resistant only to β-lactams and to tetracyclines. In contrast, the presence of multiresistant LA-MRSA was also seen with the four remaining isolates showing resistance to several classes of agents. The identification of multi-resistant strains among isolates from milk samples is alarming and requires strict monitoring schemes.

Among the LA-MRSA isolates of this study no isolate harbored an enterotoxin gene. LA-MRSA isolates have been shown to harbor very rarely enterotoxin genes (Kadlec et al., 2009; Argudín et al., 2013). Thus, the result of this study fits well to the known situation. Differences in genes associated to virulence are in general alarming. The acquisition of virulence genes is a worrying possibility for this type of MRSA. LA-MRSA can adapt to different hosts and seem to be very flexible in the acquisition of resistance genes (Kadlec et al., 2012). In case they make use of their genomic flexibility and virulence properties start to increase, such isolates would have another big advantage. In this study still a favorable situation is seen with only very few differences in global regulator genes and in genes associated to virulence.

## Conclusion

This study showed a high detection rate of staphylococci in bovine quarter milk samples. Among the staphylococcal isolates, LA-MRSA was rarely detected. All 14 LA-MRSA belonged to CC398, whereas only 11/358 MSSA (3.1%) belonged to this clonal complex.

## Author Contributions

All authors participated in the study and provided additions and corrections to the manuscript. KK wrote the first draft.

## Conflict of Interest Statement

ME and TP were employed by the Dairy Herd Consulting and Research Company (MBFG). The remaining author declares that the research was conducted in the absence of any commercial or financial relationships that could be construed as a potential conflict of interest, however KK is meanwhile employed by the MBFG.
